# Urinary Metabolomics as a Window into Occupational Exposure: The Case of Foundry Workers

**DOI:** 10.3390/jox16010014

**Published:** 2026-01-15

**Authors:** Michele De Rosa, Silvia Canepari, Giovanna Tranfo, Ottavia Giampaoli, Adriano Patriarca, Agnieszka Smolinska, Federico Marini, Lorenzo Massimi, Fabio Sciubba, Mariangela Spagnoli

**Affiliations:** 1 Department of Chemistry, Sapienza University of Rome, Piazzale Aldo Moro 5, 00185 Rome, Italy; michele.derosa@uniroma1.it (M.D.R.); adriano.patriarca@uniroma1.it (A.P.); federico.marini@uniroma1.it (F.M.); 2 NMR-Based Metabolomics Laboratory, Sapienza University of Rome, Piazzale Aldo Moro 5, 00185 Rome, Italy; ottaviagiampaoli@gmail.com (O.G.); fabio.sciubba@uniroma1.it (F.S.); 3 Department of Environmental Biology, Sapienza University of Rome, Piazzale Aldo Moro 5, 00185 Rome, Italy; silvia.canepari@uniroma1.it (S.C.); l.massimi@uniroma1.it (L.M.); 4 Department of Medicine, Epidemiology, Environmental and Occupational Hygiene (INAIL), Via Fontana Candida 1, 00078 Monte Porzio Catone, Italy; g.tranfo@inail.it; 5 Faculty of Health, Medicine and Life Sciences, Pharmacology and Toxicology, Maastricht University, Universiteitssingel 50, 6229 ER Maastricht, The Netherlands; a.smolinska@maastrichtuniversity.nl; 6 Nutrition and Translational Research in Metabolism (NUTRIM), School of Nutrition & Translational Research in Metabolism, Maastricht University, Universiteitssingel 50, 6229 ER Maastricht, The Netherlands; 7 Interdepartmental Center of Applied Sciences for the Protection of the Environment and Cultural Heritage (CIABC), Sapienza University of Rome, Piazzale Aldo Moro 5, 00185 Rome, Italy

**Keywords:** NMR spectroscopy, untargeted metabolomics, multivariate statistical analysis, occupational exposure, workers biomonitoring, urinary profiles

## Abstract

Foundries represent complex exposure scenarios where metals, particulate matter, and combustion by-products coexist, posing potential cumulative biological effects. Urinary metabolic profiles from 64 foundry workers and 78 residents living in surrounding areas were investigated using multivariate statistical modeling. Differences in urinary metabolite patterns were observed between the two groups, including lower levels of several amino acids (e.g., valine, alanine, tyrosine, and tryptophan) and tricarboxylic acid intermediates (e.g., citrate and succinate), together with higher levels of selected branched-chain amino acid catabolites (e.g., 3-hydroxyisobutyrate and erythro-2,3-dihydroxybutyrate) in workers. Variations in gut microbiota-related metabolites, such as phenylacetylglycine and *p*-cresol sulphate, were also detected. Based on these metabolic patterns, potential molecular mechanisms related to energy metabolism, oxidative stress and host–microbiome interaction are discussed as interpretative hypotheses. The comparison between workers and residents was interpreted, taking into account differences in demographic and lifestyle characteristics between groups. Overall, the results indicate that occupational exposure in foundries is associated with measurable differences in urinary metabolic profiles, demonstrating that the applied NMR-based metabolomic strategy is capable of capturing early biological effects and supporting its potential as a non-invasive and holistic biomonitoring tool for evaluating the health impact of complex occupational exposures.

## 1. Introduction

The European foundry industry ranks third globally in the production of ferrous metals and second for non-ferrous metals, with an estimated annual output of 11.7 million tons for ferrous and 2.8 million tons for non-ferrous alloys [1]. Within this framework, Italy represents the second-largest foundry-producing country in Europe, after Germany, and the ninth worldwide. Together, Italy and Germany account for nearly 70% of the total European casting production. In 2023, the Italian foundry sector comprised 1038 active plants, employing approximately 24,000 workers [2]. The majority of Italian foundries (866) focus on non-ferrous metals, while 172 are dedicated to ferrous metals (81% cast iron and 19% steel). Despite their smaller number, ferrous foundries generate significantly higher average revenues (€10.8 million) compared to non-ferrous foundries (€4.9 million), and around 80% of all foundries are located in Northern Italy [2]. Foundry work involves different processing phases ranging from the molding of metals and alloys through melting to pouring the liquid into molds, and solidifying it to obtain the desired shape, as one of the oldest known techniques for metal shaping. This variety of operations generates an exposure scenario that is, to say the least, complex and involves numerous airborne contaminants. In this context, workers are potentially exposed to hazardous substances including sulfur dioxide (SO_2_), carbon monoxide (CO), nitrogen oxides (NO_x_), iron-sulfur compounds, and heavy metals such as manganese, cadmium, nickel, chromium, copper, molybdenum, and lead [3]. Some of these metals are essential micronutrients, such as iron, copper and zinc, but become toxic when present in excessive amounts. Others, like cadmium, lead, and hexavalent chromium, are non-essential and pose severe health hazards even at low concentrations. For example, chromium (VI) is a known Group 1 carcinogen and can cause respiratory irritation, liver and spleen toxicity, and skin necrosis [4,5], while nickel has been linked to genotoxicity, immunotoxicity, hepatotoxicity, nephrotoxicity, and multiple forms of cancer [6,7]. Excessive manganese inhalation is associated with pulmonary damage and neurological effects, while molybdenum exposure has been correlated with reproductive toxicity and nervous system impairment, especially in occupational settings [8,9]. Even essential elements like iron can lead to acute gastrointestinal symptoms or chronic effects such as metabolic acidosis, liver necrosis, and death if absorbed in large quantities [10]. Even when limiting the analysis to a simplified and exclusively qualitative consideration of the aforementioned elements and their different mechanisms of action [11], the difficulty of obtaining a comprehensive risk assessment becomes evident. In the described context, metabolomics represents a powerful tool for the evaluation of the health effects of airborne particulate matter, providing a holistic view of the state of an organism subjected to stressful conditions. This is particularly relevant in occupational environments like that under examination, where workers are exposed to dynamic mixtures of xenobiotics that may interact synergistically. The potential of *omics* approaches has already been presented in several studies in different fields [12,13,14], but despite this, their application in occupational hygiene settings still remains limited. In light of this, the present work aims to characterize the urinary metabolome of workers employed in a foundry in comparison with a group of residents living in areas surrounding the factory, in order to identify potential metabolic alterations associated with occupational exposure.

## 2. Materials and Methods

### 2.1. Study Design

The authors enrolled sixty-four foundry workers and seventy-eight residents living in the areas surrounding the factory. Participants were recruited on a voluntary basis. Eligible subjects were adults (≥18 years) in good general health, without known metabolic, renal, or chronic inflammatory diseases. Only individuals who met the inclusion criteria and provided written informed consent were enrolled. A convenience sampling approach was adopted, based on voluntary participation among the two predefined groups (workers and residents). No randomization procedures were applied during recruitment, as the study aimed to compare occupationally exposed individuals with a control population living in the surrounding area. For each subject, a first-morning, midstream, one-spot urine sample was collected following standardized instructions to minimize intra-individual variability. Samples were immediately refrigerated after collection and transported to the laboratory. Upon arrival, urine samples were aliquoted and stored at –80 °C until metabolomics profiling. Metabolomic analysis was performed using high-resolution NMR spectroscopy. This study was approved by the Ethical Committee “CET Lazio Area 2”, study protocol: “BRIC ID 52”, protocol ID:113.24 CET2 ptv. All experiments were conducted according to the Declaration of Helsinki and following the International Code of Ethics for Occupational Health Professionals, published by the International Committee of Occupational Health (ICOH). The information gathered was used as aggregate data, with no risk of individual identification.

### 2.2. Sample Preparation and NMR Experiments

All samples were pretreated according to a previously described protocol [15] aimed at obtaining high-throughput analysis with minimal sample alteration and maximum retention of metabolic information content, in accordance with metabolomics requirements. Briefly, the protocol included a centrifugation step (15 min, 3500 rpm, 4 °C) to remove cells, debris, and other particulate matter that could interfere with the spectral quality; a dilution of the supernatant (400 µL) with phosphate buffer (200 µL, pH = 7) for pH adjustment, and the addition of an internal standard (3-(trimethylsilyl) propionic-2,2,3,3-d4 acid sodium salt—TSP—in D_2_O) solution for chemical shift referencing. Details are provided elsewhere [16]. All NMR spectra were acquired using a 600 MHz JEOL JNM-ECZR spectrometer (JEOL Ltd., Tokyo, Japan). Compound identification was carried out by acquiring both homonuclear (^1^H-^1^H TOCSY) and heteronuclear (^1^H-^13^C HSQC, ^1^H-^13^C HMBC) 2D-NMR experiments on selected samples and cross-checking correlation frequencies with an online freely available database [17,18]. A proton NMR spectrum of a urinary sample with the identified metabolites is shown for illustrative purposes in Supplementary Figure S1. Furthermore, mean spectra were calculated to provide a representative overview of group-level metabolic profiles, reducing inter-individual variability (Supplementary Figure S2). Experimental parameters were set analogously to those previously reported [16].

### 2.3. Spectra Preprocessing and Statistical Analysis

All spectra were manually preprocessed by window function application (lb = 0.3), Fourier Transform, phase correction, baseline correction and chemical shift referencing (against TSP singlet at 0.00 ppm). Any baseline humps, caused by imperfect suppression of the solvent signal or by digitalization noise, were eliminated by applying the baseline correction through the FID reconstruction (BCFR) procedure. The entire preprocessing was carried out using ACD Labs software v.12.0 (Advanced Chemistry Development, Inc., 8 King Street East, Toronto, ON, Canada). After a further baseline refinement, all spectra were subjected to alignment [19], adaptive intelligent binning [20] and numerical normalization [21]. Raw binned ^1^H-NMR spectral intensities used for multivariate statistical analyses are reported in Supplementary Table S1. Unsupervised Random Forest (URF) and supervised PLS-LDA were applied to the entire dataset after log-transformation and auto-scaling to highlight any differences between the two groups (exposed workers and residents). For URF, 50 iterations, 1500 trees and 8 samples in the final leaves were chosen as tuning parameters. The PLS-LDA model was validated by the repeated double cross-validation method, using accuracy, sensitivity, specificity and correct classification rate as model validation parameters. The significance of each parameter was assessed by permutation tests. Significant variables for discrimination were selected based on weights along the first canonical variate, only taking into account those whose sign remained unchanged during the validation procedure. MATLAB 2024b software (Natick, MA, USA: The MathWorks Inc.) was employed for all analyses with in-house written functions.

## 3. Results

### 3.1. Population Description

During sample collection, each participant completed a structured questionnaire, which was provided together with the informed consent form. The survey was designed to gather detailed information on the subject’s health status at the time of recruitment, drug intake, potential sources of exposure other than the one under investigation, dietary habits, alcohol consumption, smoking status, and general lifestyle factors. These data were used to characterize the study population and to identify potential confounders. Main characteristics of the enrolled subjects are summarized in Table 1. A comparison of the main demographic and lifestyle variables between workers and residents showed that the largest differences were related to a higher mean age in the resident group and a higher prevalence of smokers among workers. In contrast, a high degree of homogeneity between the two groups was observed for sex distribution, alcohol consumption, and BMI.

### 3.2. Urinary Metabolomic Profiles

**Figure 1:**
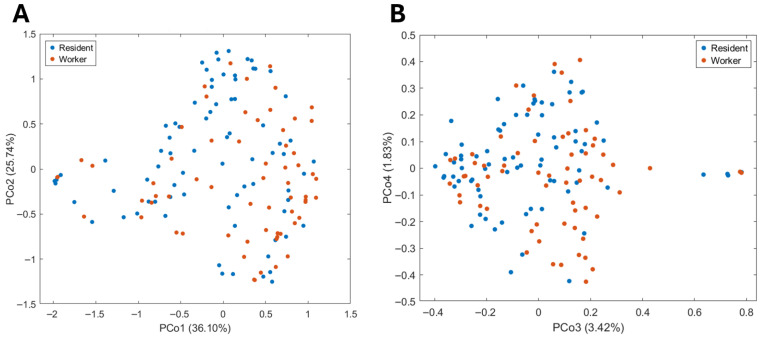
Scores plots obtained from Unsupervised Random Forest analysis. (**A**) PCo1 versus PCo2 score plot. (**B**) PCo3 versus PCo4 score plot. Residents are shown in blue and workers in orange; percentages on the axes indicate the explained variance by each principal coordinate.

Figure 1 shows score plots obtained from PCoA applied to the dissimilarity matrix generated by URF. From these plots, it can be observed that no spontaneous grouping of samples happens based on exposure.

**Figure 2:**
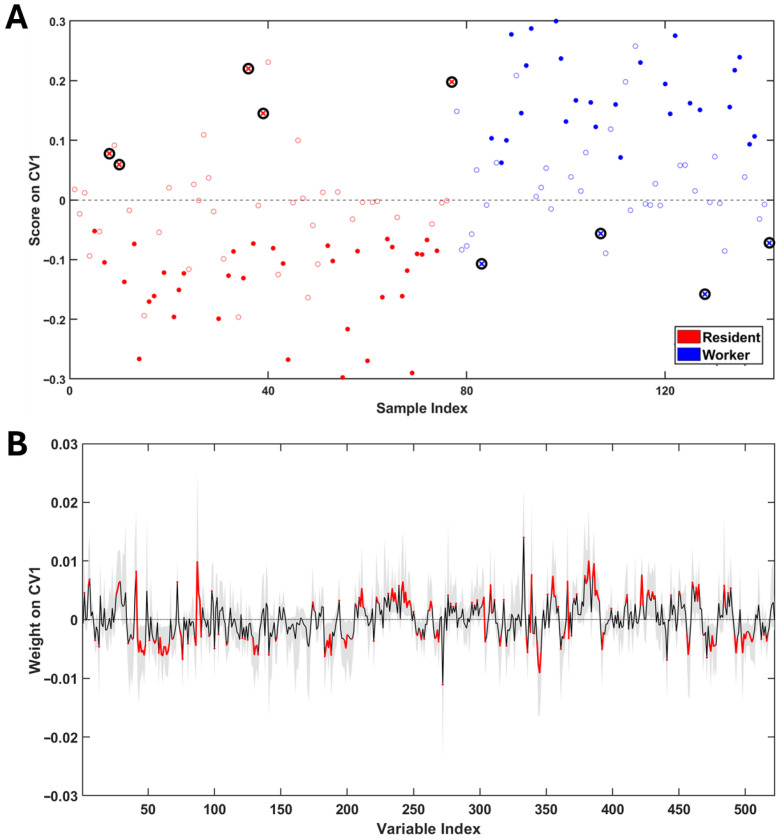
(**A**) PLS-LDA score plot for the comparison between residents (in red) and workers (in blue). Filled dots represent samples that were always correctly classified during cross-validation cycles. Empty dots are samples whose confidence interval was 0 during cross-validation. Finally, the samples represented by black circled crosses are those that are systematically misclassified. (**B**) PLS-LDA weights along the first canonical variate. The black line represents the mean weight profile, and the grey shaded area indicates the 95% confidence interval for each variable. Segments highlighted in red correspond to variables whose confidence interval bounds do not cross the threshold of 0 and that are considered significant for discrimination.

This behavior can be interpreted as affirming that the main source of dissimilarity between samples does not derive from occupational exposure. Before proceeding with the supervised analysis, it was decided to evaluate whether some plausible confounding factors impacted the homogeneity of the investigated population. Details are reported in Supplementary Materials. In brief, what emerges is that none of the considered plausible confounding factors impact sample grouping in unsupervised settings. This observation allows us to reasonably conclude that the examined population is characterized by an acceptable degree of homogeneity and the greatest dissimilarity between the samples lies in interindividual biological variability. Based on this assumption, no samples were excluded from further analysis. Subsequently, with the aim of focusing only on the effects possibly related to occupational exposure, a PLS-LDA discriminant model was built (Figure 2A,B). The model showed good validation parameters with an accuracy of 73 ± 2%, a sensitivity of 67 ± 3%, a specificity of 78 ± 3% and a correct classification rate of 72 ± 2%. Significance of each parameter was assessed by a permutation test (significance threshold *p*-value < 0.05).

From the analysis of the weights along CV1, the following significant variables for the discrimination of the exposed group (workers) emerged: leucine (Leu), lysine (Lys), acetate (AA), N-acetylglutamine (N-AcGln), p-cresol sulphate (p-CrS), creatine (Crt), tyrosine (Tyr), unknown pyrimidine and trigonelline (Trig). Conversely, urinary concentrations of valine (Val), 4-hydroxyphenylacetate (4-HPA), 1-methylnicotinamide (1-MNA), isoleucine (Ile), phenylacetylglycine (PAG), 4-hydroxybenzoate (4-HBz), 3-hydroxyisobutirate (3-HIBA), tryptophan (Trp), pseudouridine (PSI), erythro-2,3-dihydroxybutyrate (Erythro-2,3-DHBA), 3-hydroxy-3-methylbutyrate (3-H-3-MBA), hippurate (Hipp), succinate (SA), pyro-glutamate (pyro-Glu), citrate (CA), taurine (Tau), creatinine (Crtn) and furoylglycine exhibited negative weights, indicating an association with the residents group. The results presented are summarized in Table 2.

## 4. Discussion

Based on the observed differences in metabolomic profiles, it is possible to propose some hypotheses on the effect of exposure on the organism. First, attention can be focused on a potential alteration of amino acid metabolism. This finding is supported by the alterations observed in urinary levels of both aromatic and aliphatic free amino acids, as well as some branched-chain amino acid catabolites such as 3-HIBA, erythro-2,3-DHBA, and 3-H-3-MBA. Recent metabolomic studies have revealed that heavy metals can profoundly interfere with amino acid metabolism [22,23]. This interference occurs through several interconnected processes. Heavy metals such as cadmium, mercury, and lead are known to bind to thiol groups within the active sites of enzymes [24], leading to the inhibition of key enzymes involved in amino acid synthesis and catabolism, such as glutathione reductase and δ-aminolaevulinic acid dehydratase (ALAD), the latter being particularly susceptible to lead [25,26]. The resulting enzymatic inhibition disrupts amino acid homeostasis and compromises cellular redox balance. Furthermore, cellular stress caused by heavy metal exposure triggers an increased turnover of proteins as part of the cellular repair response, aiming to replace or degrade damaged and misfolded proteins through mechanisms such as the ubiquitin–proteasome system [27,28]. In humans, metallothioneins, cysteine-rich metal-binding proteins, play a central role in modulating the cellular response to metal exposure [29,30]. These proteins sequester toxic metals like cadmium and mercury, limiting their interaction with critical biomolecules and mitigating oxidative damage. However, when metal exposure exceeds the binding capacity of metallothioneins, free metal ions accumulate and exert cytotoxic effects. Additionally, heavy metals can alter key metabolic pathways such as the tricarboxylic acid (TCA) cycle and the kynurenine pathway, leading to the accumulation of neurotoxic intermediates like quinolinic acid and a reduction in protective metabolites such as kynurenic acid. Overall, the interactions between heavy metals and amino acid metabolism are complex and multifaceted, involving enzymatic inhibition, compensatory metabolic shifts, oxidative modifications of amino acids, and insufficient detoxification responses, all of which contribute to their cumulative toxicity [31,32]. Regarding TCA cycle impairment, this aspect is also reflected in the presented results, particularly by the observed decrease in key intermediates such as citrate and succinate. This depletion can be interpreted through several interconnected mechanisms. One possibility is the direct inhibition of TCA enzymes by airborne xenobiotics, including heavy metals, which are known to interfere with enzyme activity by binding to essential cofactors or catalytic residues, often targeting sulfhydryl (-SH) groups [33]. Additionally, a broader compromise of mitochondrial function may be at play. Heavy metals such as cadmium and arsenic have been shown to impair mitochondrial respiration and ATP production, thereby disrupting energy metabolism at its core [34]. This mitochondrial dysfunction is often exacerbated by redox imbalance, as heavy metals promote excessive ROS production, damaging mitochondrial DNA, lipids, and proteins, further impairing oxidative phosphorylation and metabolic flux through the TCA cycle. Alternatively, the observed decrease in TCA intermediates might reflect a metabolic reprogramming in response to stress. In particular, intermediates such as citrate and α-ketoglutarate can be diverted toward alternative biosynthetic or regulatory pathways, including lipid synthesis, amino acid transamination, or antioxidant responses involving glutathione metabolism. This redirection is a hallmark of cellular adaptation under oxidative or toxic stress, whereby metabolic flexibility enables cells to buffer damage and restore homeostasis [35].

Another noteworthy aspect emerging from metabolic profiling is the significant variation in metabolites such as 4-HPA, hippurate, and 4-HBz, which are derived from the microbial metabolism of dietary polyphenols. Additionally, metabolites like PAG and p-cresol sulfate, linked to phenylalanine metabolism, are also produced through the action of intestinal bacteria. The modulation of these compounds suggests potential morpho-functional alterations of the gut microbiota following exposure to environmental contaminants. This hypothesis is consistent with growing evidence that many xenobiotics, including heavy metals, solvents, and particulate matter, can directly affect gut microbial composition or indirectly alter the host–microbiota interaction by modulating immune, inflammatory, or metabolic pathways [36]. The gut microbiome plays a crucial role in the biotransformation of endogenous and exogenous substances, and its disruption, commonly referred to as dysbiosis, has been associated with a wide array of pathologies, ranging from metabolic syndrome and neurodegenerative diseases to gastrointestinal and hepatic disorders [37].

In particular, dysbiosis has been implicated both as a consequence and a driver of systemic toxicity, forming a feedback loop that exacerbates physiological imbalances. Several studies have demonstrated that heavy metals such as cadmium, arsenic, and lead can alter the diversity and abundance of gut microbial populations, decrease beneficial taxa (e.g., *Lactobacillus* and *Bifidobacterium*), and increase opportunistic pathogens [38,39,40,41]. These shifts can compromise gut barrier integrity, increase the production of endotoxins, and impair microbial metabolic functions, such as short-chain fatty acid synthesis or xenobiotic detoxification. Furthermore, it is essential to note that metabolites like PAG and hippurate, despite their microbial origin, are also conjugated in the liver as glycine derivatives, implicating hepatic phase II metabolism in their final biosynthesis. This dual dependency on both gut and liver function highlights the complexity of host–microbiota co-metabolism. The liver, being a primary site of xenobiotic processing, is particularly vulnerable to the toxic effects of environmental pollutants. Heavy metals, solvents, and polycyclic aromatic hydrocarbons have been shown to impair hepatic detoxification pathways, disturb bile acid metabolism, and induce oxidative stress and inflammation in hepatocytes [42,43,44]. Consequently, fluctuations in glycine-conjugated metabolites could reflect not only microbial dysbiosis but also hepatic dysfunction.

## 5. Conclusions

The present study highlights differences in urinary metabolomic profiles between foundry workers and residents from surrounding areas, as detected by an untargeted NMR-based approach. The observed variations involved amino acid-related metabolites, intermediates of central energy metabolism, and gut microbiota–derived compounds, revealing metabolic patterns that may reflect adaptive or responsive changes associated with occupational exposure. By capturing these subtle metabolic variations, the untargeted metabolomic strategy demonstrates its utility as a non-invasive tool for investigating early biological responses in complex occupational settings. This work represents one of the first applications of untargeted urinary metabolomics to assess low-dose, multifactorial exposures in foundry workers, providing a foundation for future mechanistic studies and the identification of early biomarkers of exposure.

Some limitations should be acknowledged. Sample size was limited, participation was voluntary, and only a single first morning urine sample per participant was collected, limiting generalizability, the control of potential confounders, and temporal resolution. Future studies should, therefore, aim to validate these findings in larger cohorts to strengthen the statistical power and generalizability of the results. In addition, the integration of complementary analytical platforms, such as LC–MS, GC–MS and targeted assays (e.g., oxidative stress biomarkers) could enhance metabolite coverage while also providing a deeper understanding of the biochemical mechanisms underlying occupational exposure. Moreover, combining metabolomic data with other omics layers, such as transcriptomics, proteomics, or metagenomics, together with detailed exposure assessment, could facilitate the identification and validation of specific biomarkers of early biological effect, thus strengthening the mechanistic interpretation of exposure–response relationships in occupational settings.

## Figures and Tables

**Table 1:** Characteristics of enrolled subjects.

	Age (MEAN ± SD)	Males (N)	Females (N)	Smokers (N)	Alcohol Consumption (N)	BMI (Mean) (Min-Max)
Workers	47 ± 8	38	26	24	44	24.4 (18.4–31.2)
Residents	57 ± 10	41	36	7	58	25.0 (18.4–39.4)

**Table 2:** Metabolites contributing to group discrimination in the PLS-DA model.

Metabolite	CV1 Weight (Sign)	Association with Group	Metabolite Class	Main Biological Role/Origin
Leucine (Leu)	+	workers	Branched-chain amino acid	Protein synthesis/turnover, energy metabolism
Isoleucine (Ile)	–	residents	Branched-chain amino acid	Protein synthesis/turnover, energy metabolism
Valine (Val)	–	residents	Branched-chain amino acid	Protein synthesis/turnover, energy metabolism
Lysine (Lys)	+	workers	Essential amino acid	Protein synthesis/turnover, energy metabolism
Tyrosine (Tyr)	+	workers	Aromatic amino acid	Neurotransmitter and hormone precursor, protein synthesis
Tryptophan (Trp)	–	residents	Aromatic amino acid	Hormone precursor, Protein synthesis/turnover, energy metabolism
N-acetylglutamine (N-AcGln)	+	workers	Amino acid derivative	Energy metabolism, detoxification
Acetate (AA)	+	workers	Short-chain fatty acid	Gut microbial fermentation, energy metabolism
Citrate (CA)	–	residents	Organic acid	Energy metabolism
Succinate (SA)	–	residents	Organic acid	Energy metabolism
3-Hydroxyisobutyrate (3-HIBA)	–	residents	Organic acid	Valine catabolism
Erythro-2,3-dihydroxybutyrate (Erythro-2,3-DHBA)	–	residents	Organic acid	Branched-chain amino acids catabolism
3-Hydroxy-3-methylbutyrate (3-H-3-MBA)	–	residents	Organic acid	Branched-chain amino acids catabolism
*p*-Cresol sulfate (p-CrS)	+	workers	phenyl sulfate	Microbiota-derived uremic toxin, Aromatic AA catabolism
Creatine (Crt)	+	workers	Nitrogen based compound	Phosphocreatine shuttle, muscle metabolism
Creatinine (Crtn)	–	residents	Cyclic amide	Muscle metabolism, renal excretion
Unknown pyrimidine	+	workers	Nucleotide related metabolite	Nucleic acid metabolism (putative)
Trigonelline (Trig)	+	workers	Alkaloid	Dietary origin (coffee), niacin metabolism
4-Hydroxyphenylacetate (4-HPA)	–	residents	Phenolic compound	Gut microbial metabolism of tyrosine
Phenylacetylglycine (PAG)	–	residents	Host–microbiome co-metabolite	Microbial amino acid metabolism
Hippurate (Hipp)	–	residents	Host–microbiome co-metabolite	Microbial metabolism of dietary polyphenols
Pyroglutamate (pyro-Glu)	–	residents	Amino acid derivative	Glutathione metabolism
Taurine (Tau)	–	residents	Sulfur-containing amino acid	Osmoregulation, bile acid conjugation
1-Methylnicotinamide (1-MNA)	–	residents	nicotinamide	Vitamin B3 metabolite, vasoprotective/anti-inflammatory action
Furoylglycine	–	residents	Glycinate compound	Gut microbial metabolism of dietary compounds
4-Hydroxybenzoate (4-HBz)	–	residents	Phenolic acid	Microbial degradation of aromatics
Pseudouridine (PSI)	–	residents	Modified nucleoside	RNA turnover

## Data Availability

The original contributions presented in this study are included in the article/Supplementary Material. Further inquiries can be directed to the corresponding author.

## Supplementary Materials

The following supporting information can be downloaded at https://www.mdpi.com/article/10.3390/jox16010014/s1. Figure S1: ^1^H-NMR spectra with resonance assignments; Figure S2: Mean urinary ^1^H-NMR spectra calculated for each study group; Figure S3: Score plots obtained from Unsupervised Random Forest analysis color-coded based on plausible confounding factors; Table S1: Raw binned ^1^H-NMR spectral intensities used for multivariate statistical analyses.
