# Supplementary material for: Urinary Metabolomics as a Window into Occupational Exposure: The Case of Foundry Workers
Source: J Xenobiot. 2026 Jan 15;16(1):14. doi: 10.3390/jox16010014 (PMC12821577; doi:10.3390/jox16010014)
Supplement: Supplementary file 1 [file jox-16-00014-s001.zip › jox-4054261-Supplementary.pdf]

Journal of *Xenobiotics*

MDPI

Supplementary Materials: Urinary metabolomics as a window on occupational exposure: The case of foundry workers

Michele De Rosa, Silvia Canepari, Giovanna Tranfo, Ottavia Giampaoli, Adriano Patriarca, Agnieszka Smolinska, Federico Marini, Lorenzo Massimi, Fabio Sciubba and Mariangela Spagnoli

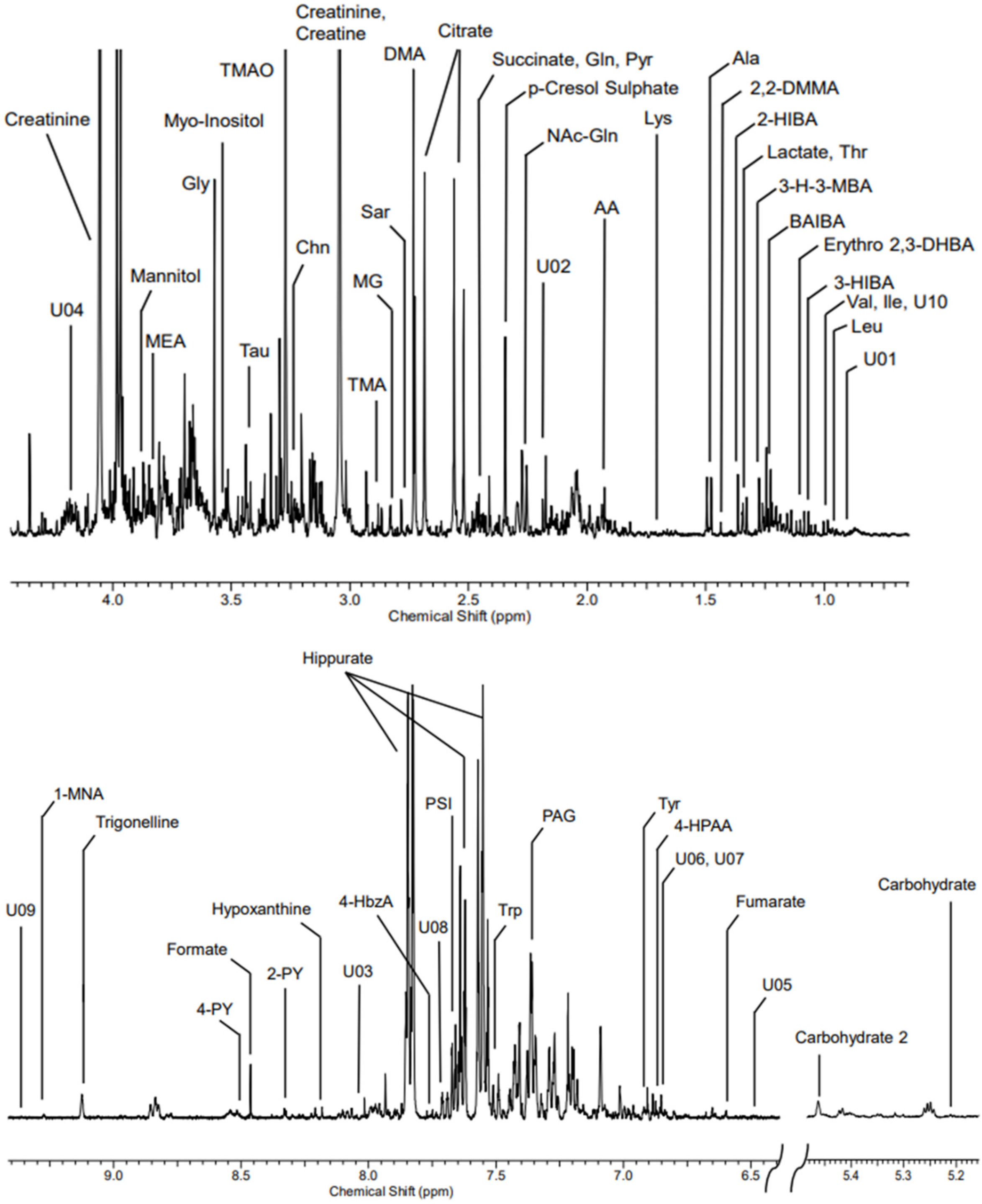

Figure S1. ^{1}H-NMR spectrum of typical urine sample in regions **(up)** 0.5 - 4.4 ppm and **(bottom)** 5.2 – 9.5 ppm. Resonances are referenced to TSP singlet at 0.00 ppm (not shown). The dark regions between 4.5 – 5.5 ppm and 5.4 – 6.5 ppm correspond to the resonances of water and urea respectively. List of abbreviations: leucine (Leu), valine (Val),

isoleucine (Ile), 3-hydroxyisobutyrate (3-HIBA), erythro-2,3-dihydroxubutyrate (erythron 2,3-DHB), 3-aminoisobutanoic acid (BAIBA), 3-hydroxy-3-methylbutyrate (3-H-3-MBA), threonine (Thr), 2-hydroxyisobutyrate (2-HIBA), 2,2-dimethylmalonate (2,2-DMMA), alanine (Ala), lysine (Lys), acetate (AA), N-acetylglutamine (NAc-Gln), glutamine (Gln), pyruvate (Pyr), dimethylamine (DMA), sarcosine (Sar), methylguanidine (MG), trimethylamine (TMA), choline (Chn), trimethylamine-N-oxide (TMAO), taurine (Tau), glycine (Gly), ethylamine (MEA), 4-hydroxyphenylaetate (4-HPAA), tyrosine (Tyr), phenylacetylglycine (PAG), tryptophane (Trp), pseudouridine (PSI), 4-hydroxybenzoate (4-HBz), N-methyl-2-pyridone-5-carboxamide (2-PY), N-methyl-4-pyridone-3-carboxamide (4-PY), 1-methylnicotinamide (1-MNA), unknown compound (U).

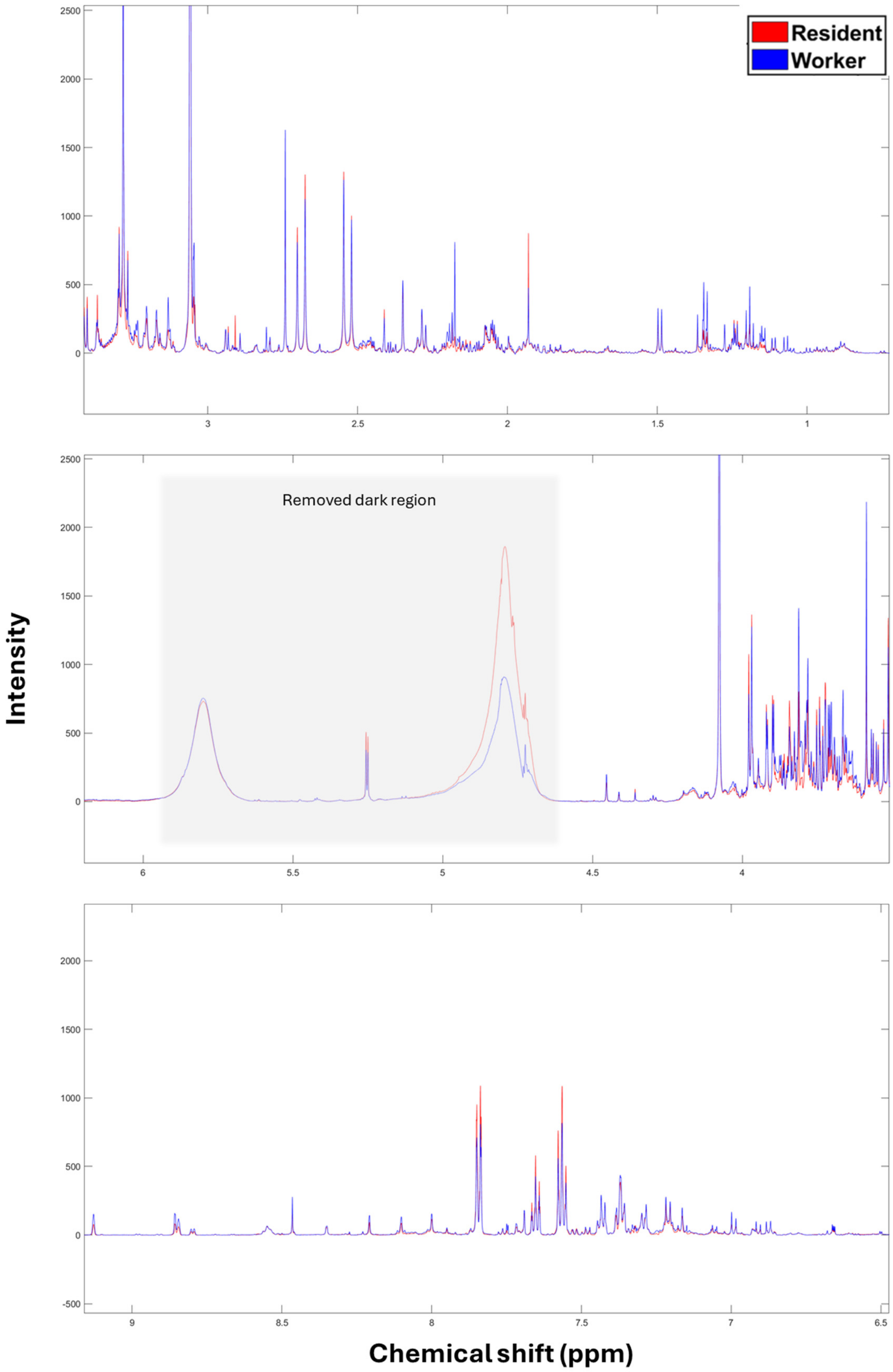

Figure S2. Mean urinary ¹H-NMR spectra calculated for each study group, showing the average metabolic profiles across all subjects within each category. Resonances are referenced to TSP singlet at 0.00 ppm (not shown).

Assessment of potentially confounding factors

It is known that metabolomics could be affected potentially by numerous confounders related to age, sex, drugs assumption, alcohol intake, smoke, diet and more in general lifestyle habits of subjects enrolled in the study. Completely eliminating such confounding factors is extremely challenging in observational studies that involve voluntary recruitment of participants. On the other hand, excessive stratification during statistical analysis, although remains the best strategy for experimental settings based on single-biomarker approach; when using omics platforms, where a large sample size is required to achieve good statistical robustness, could not be the right way to proceed. It is important to carefully consider excluding certain subjects from the analysis. For this reason, in this study the impact of possible confounding factors was assessed through the ability of each factor to generate spontaneous clustering of the data. In particular, demographic information on the study population, obtained through detailed surveys administered during enrollment, were extracted and coded as different levels of each factor and then unsupervised random forest analysis was applied. Gender, age, smoking and alcohol or drugs intake were considered as confounding factors. Age and drugs consumption was considered in combination given their potential correlation. Alcohol consumption was assessed through a self-administered questionnaire in which participants were asked to indicate whether they were *abstainers*, *occasional*, or *habitual drinkers*, and to report their average number of alcoholic drinks per day. Regarding drugs, although participants reported various types of medications, their number and heterogeneity did not allow for subgroup analysis by specific drug type. Therefore, drug consumption was considered as a binary variable (users vs. non-users) to assess its potential influence on urinary metabolic profiles. In order to verify how much these spurious factors impact the dissimilarity between samples, the score plots obtained by URF are reported represented with a different color codes in figure S2. What emerges from this representation is that none of the considered plausible confounding factors impact samples grouping. This observation allows to reasonably affirm that the examined population is characterized by an acceptable degree of homogeneity and the greatest dissimilarity between the samples lies in interindividual biological variability. Obviously, this assumption is not sufficient to state that the analysis performed is not affected by any confounding factors, however, in our opinion it represents a conservative strategy to avoid impacting the sample size available and consequently the statistical robustness of the results presented.

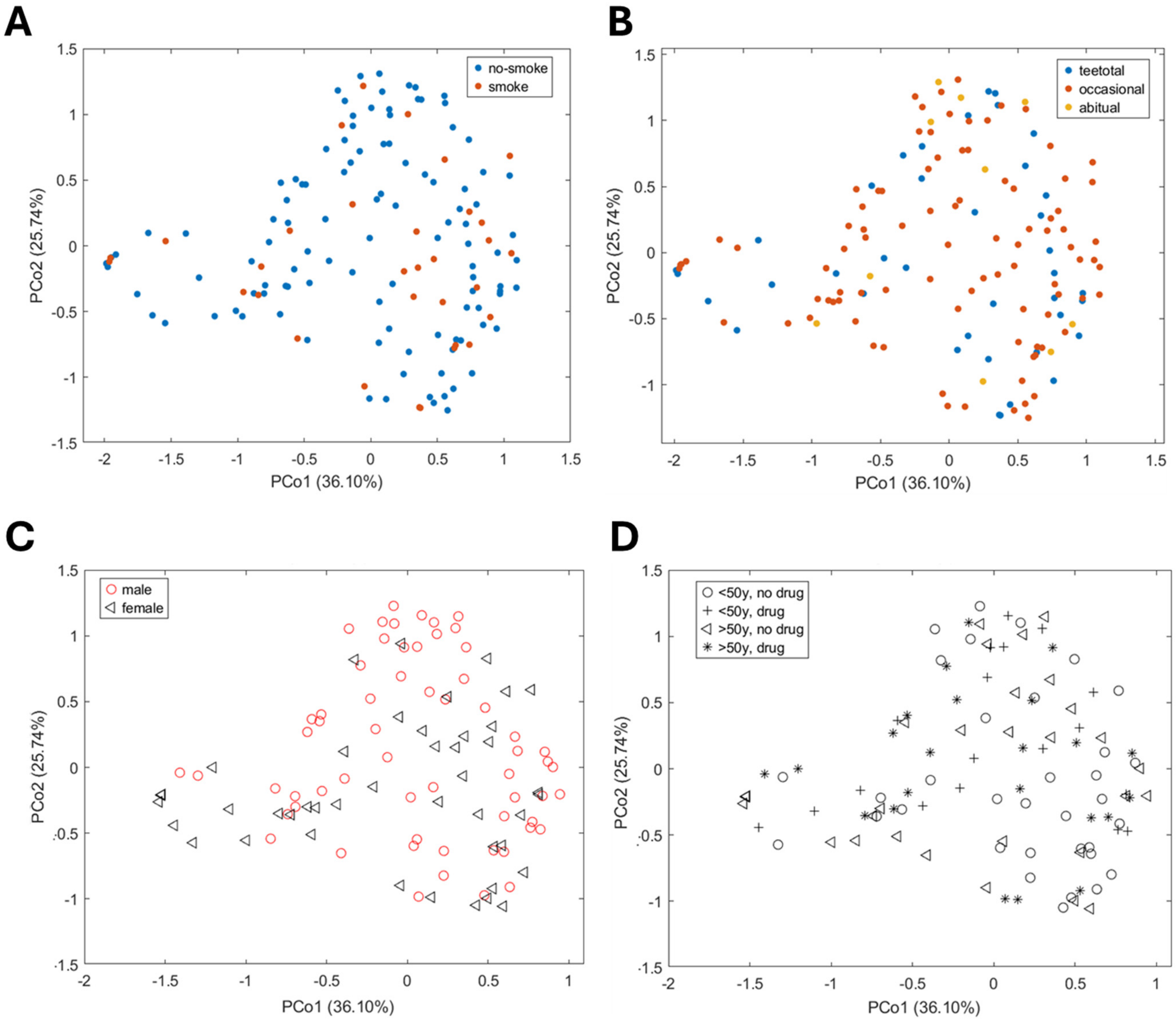

Figure S3. Score plots obtained from Unsupervised Random Forest analysis color-coded based on plausible confounding factors. (A) PCo1 versus PCo2 sample scores color coded according to smoke habits (no-smoke vs. smoke). (B) PCo1 versus PCo2 sample scores color coded according to alcohol consumption (teetotal, occasional, habitual). (C) PCo1 versus PCo2 sample scores color coded according to sex (males vs. females). (D) PCo1 versus PCo2 sample scores color coded according to the combination of age and drugs intake (under 50 years-no drug intake, under 50 years-drug intake, over 50 years-no drug intake, over 50 years-drug intake).
